# Harnessing Molecular Insights for Translational Impact: Highlights from the Special Issue Titled “New Insights in Translational Bioinformatics”

**DOI:** 10.3390/ijms26020579

**Published:** 2025-01-11

**Authors:** Camelia Quek

**Affiliations:** 1 Melanoma Institute Australia, Sydney, NSW 2065, Australia; camelia.quek@sydney.edu.au; 2 Faculty of Medicine and Health, The University of Sydney, Sydney, NSW 2006, Australia; 3 Charles Perkins Centre, The University of Sydney, Sydney, NSW 2006, Australia

## 1. Introduction and Scope of the Special Issue

The field of translational bioinformatics is rapidly evolving, driving the convergence of molecular sciences and computational methods with their applications in industrial and clinical practice. This Special Issue, “New Insights in Translational Bioinformatics”, in the *International Journal of Molecular Sciences* exemplifies the interdisciplinary spirit of molecular research, showcasing research that bridges fundamental molecular science with applied perspectives in society. The main goal in this Special Issue is to feature studies that not only advance theoretical understanding but also demonstrate translational potential in addressing pressing global challenges.

**Figure 1:**
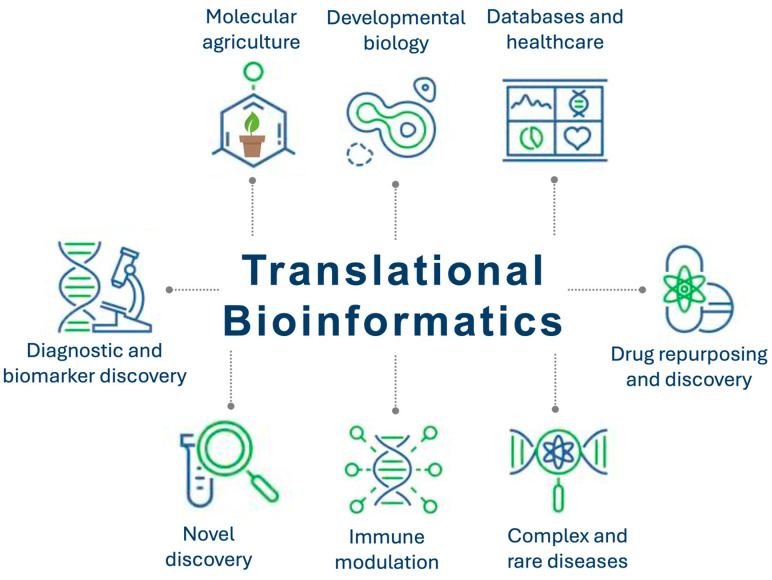
An overview of the applications in translational bioinformatics. The diagram illustrates the diverse applications of translational bioinformatics across key domains, including agricultural biotechnology, developmental biology, databases, healthcare, drug repurposing, drug discovery, complex diseases, immune modulation, novel discovery, biomarker development, and diagnostics. These diverse domains underscore the pivotal role of translational bioinformatics in bridging fundamental research and real-world solutions.

In this Special Issue, the collection contains nine original research articles that address critical challenges in diverse domains, contributing significantly to our understanding of molecular mechanisms and their applications in disease diagnosis, treatment, and fundamental biological processes (Figure 1). This editorial summarises the contributions to this Special Issue, explores their broader implications, and highlights potential future directions, thereby providing a comprehensive overview that situates these studies within the broader context of molecular science and its transformative potential.

## 2. Highlights of the Special Issue

### 2.1. Cancer Research and Therapeutics

In the context of cancer, the tumour microenvironment (TME) is a complex ecosystem composed of a variety of molecular and cellular components surrounding the tumour [1,2]. The approach of combining interdisciplinary fields allows one to understand the interconnectedness that brings deeper insights to advance our knowledge of cancer biology, diagnostics, and potential cancer therapies [3,4].

#### 2.1.1. Repurposing Sitagliptin for Colorectal Cancer

Colorectal cancer (CRC) remains a highly lethal malignancy with poor outcomes, often driven by cancer stem cells (CSCs) and treatment resistance. Shih and Wu et al. investigated the potential of repurposing sitagliptin, a dipeptidyl peptidase-4 inhibitor commonly used in diabetes treatment, as a therapeutic agent for CRC [5]. The study hypothesised that sitagliptin could target the *CD24/SOX4* signalling hub, which plays a crucial role in CSC maintenance and the tumour microenvironment, to improve the effectiveness of CRC therapies and provide a new therapeutic avenue for an existing drug. The research employed a multidisciplinary approach that involves a combination of bioinformatics, molecular docking, and in vitro experiments. The study highlighted that increased expression of *CD24* and *SOX4* was associated with poor prognosis in CRC. The CD24-positive cancer-associated fibroblasts were also implicated in tumour progression, highlighting a critical role for the *CD24/SOX4*-centred signalling hub. Using molecular docking, sitagliptin was shown to bind with high affinity to key molecules in this signalling pathway, suggesting its therapeutic potential. Furthermore, the in vitro experiments demonstrated that sitagliptin reduced the tumourigenic properties of CRC cells, inhibited CSC characteristics, and enhanced the efficacy of 5-fluorouracil, a standard chemotherapeutic agent for CRC. These findings suggest that sitagliptin, in combination with existing treatments, could significantly suppress CRC progression. The collective findings from this work highlight the potential to repurpose sitagliptin as a cost-effective and accessible therapeutic agent for CRC, reducing the need for novel drug development. Beyond CRC, this research opens the door for investigating sitagliptin’s effects on other cancers, broadening its clinical applications.

#### 2.1.2. Interferon Alpha Inducible Protein 6 (*IFI6*) as a Prognostic Indicator for Oesophageal Cancer

Oesophageal cancer (ESCA) is a highly aggressive malignancy with poor survival rates. Viet-Nhi et al. investigated the potential of the *IFI6* gene as a prognostic biomarker and therapeutic target, with a specific focus on its role in modulating immune responses and cancer progression [6]. The authors conducted their study using multi-omics analysis of public datasets, including The Cancer Genome Atlas (TCGA) and Gene Expression Omnibus, combined with bioinformatics approaches such as gene set enrichment analysis and gene correlation testing. *IFI6* had higher expression in tumour tissues compared to normal tissues and was strongly associated with immune pathways, including CD4+ T-cell activity and B cell receptor signalling. In line with the other published literature [7,8,9], *IFI6* was implicated in the Type I interferon signalling pathway and the defence response to viral infections, suggesting its role in modulating the immune microenvironment. Pathway enrichment analysis highlighted its involvement in critical immune-related pathways, such as the cytosolic DNA-sensing pathway and RIG-I-like receptor signalling, which are vital for detecting viral infections and activating immune responses. The outcome from this work is that *IFI6* can be used as a potential biomarker for prognosis and a target for immune-modulating therapies in ESCA. Additionally, *IFI6* could be used in diagnostic panels to identify high-risk patients and guide more aggressive treatment strategies. The overall work opens avenues for exploring *IFI6*-targeted therapies to disrupt its contribution to tumour growth and immune evasion.

#### 2.1.3. Low-Density Lipoprotein Receptor-Related Protein 1 (*LRP1*) as a Key Player in the Development of Novel Treatment Strategies in Ovarian Cancer

Ovarian cancer (OC) is the most common malignancy among gynaecological diseases worldwide. Wolde and Bhardwaj et al. aimed to explore how *LRP1*, an immune-related gene (IRG), influences the TME and its potential as a therapeutic target [10]. The research employed an integrated bioinformatics approach using datasets from TCGA and the International Cancer Genome Consortium. Statistical models were developed to evaluate the prognostic significance of *LRP1* and its associated genes and validated the nine-gene prognostic model through survival analysis and independent cohort testing. The authors identified *LRP1* as a significant component of a nine-gene prognostic model, which stratified OC patients into high-risk and low-risk groups with distinct survival profiles. In addition, *LRP1* was involved in metabolism-related pathways and immune regulation, particularly its interactions with immune checkpoint genes and components of the TME, such as immune and stromal cells. This work demonstrates that *LRP1* is not only a marker of disease severity but also actively contributes to cancer progression by modulating the TME. The potential of *LRP1* as a dual biomarker for prognosis and a target for therapeutic intervention, as well as the predictive model developed in this study, could aid in personalised treatment planning by helping clinicians identify patients who might benefit from TME-targeting therapies or closer monitoring due to their higher risk of poor outcomes. The findings highlight the importance of understanding TME heterogeneity and pave the way for innovative treatment strategies addressing both tumour cells and their supportive environment.

#### 2.1.4. ACP-BC: A Model for Accurate Identification of Anticancer Peptides

Anticancer peptides (ACPs) are small molecules with potent anticancer activities, offering advantages such as low toxicity and high biocompatibility. Sun et al. aimed to develop a novel computational model, called ACP-BC, for accurately identifying ACPs using an innovative approach that integrates biological and chemical features [11]. The study established an ACP-BC model that achieved prediction accuracies of 87% and 90% on the respective ACPs740 and ACPs240 benchmark datasets, representing improvements of 1.3% and 7% over existing methods. A key innovation was the integration of chemically derived features using the simplified molecular input line entry system notation into the model, which provided a deeper understanding of peptide properties. The model utilised three channels of feature extraction, including bidirectional long short-term memory networks for sequence-based features, chemical molecular features extracted via chemical BERT, and manually selected features such as dipeptide composition. The combination of these features enhanced the model’s ability to capture both structural and functional information about peptides. The ACP-BC model has far-reaching applications in drug discovery and development. By enabling rapid and accurate identification of ACPs, it significantly reduces the time and cost associated with experimental screening. The model opens several avenues for future research, including (i) the expansion of the dataset with more diverse peptide sequences to further enhance the model’s generalisability, (ii) the incorporation of additional features such as peptide stability, solubility, and membrane permeability to improve its applicability in clinical settings, and (iii) the extension of the model to identify peptides with activity against specific cancer types or other diseases, thereby broadening its impact. The study provides a powerful tool for designing peptide-based drugs, particularly for cancer patients resistant to standard therapies.

#### 2.1.5. Machine Learning-Driven Fluorescence In Situ Hybridisation (FISH) Probes for Distinguishing Primary Sites of Neuroendocrine Tumours

Neuroendocrine tumours (NETs) constitute a heterogeneous group of neoplasms originating from neuroendocrine cells distributed across various tissues. Accurate identification of NET primary sites is critical for optimising patient management, as pancreatic NETs (pNETs) and small bowel NETs (sbNETs) require distinct therapeutic strategies. Pietan et al. aimed to optimise the identification of primary sites in NETs using FISH probes, supported by machine learning techniques (including support vector machines and decision trees) [12]. Using a total of 44 NET samples, including 85 sbNETs and 59 pNETs, the study identified that the *ERBB2*, *MET*, and *CDKN2A* FISH probes were among the most effective biomarkers for differentiating pNETs and sbNETs. Machine learning models achieved a classification accuracy of 93.1% in distinguishing these primary sites, outperforming traditional diagnostic methods. Notably, the *ERBB2* FISH probe emerged as a highly predictive biomarker across multiple analysis models. The study also demonstrated that the imputation methods to handle missing data, such as median or k-nearest neighbour, significantly improved the robustness of the predictive models. Decision tree models provided additional insights into probe selection and feature importance, enhancing the interpretability of results. This research has significant implications for improving NET diagnostics and is particularly valuable for patients presenting with metastatic NETs of unknown origin, where treatment decisions are highly dependent on accurate site determination. Prospective clinical trials incorporating these prioritised FISH probes could assess their real-world diagnostic performance and impact on patient outcomes.

### 2.2. Rare Diseases

Advancements in translational bioinformatics have revolutionised the study of rare diseases, offering innovative solutions to overcome challenges associated with complex biological mechanisms. Microtia–atresia is a rare congenital craniofacial malformation that significantly affects physical appearance and functions such as hearing ability. Sun and Ping et al. aimed to investigate the role of the *AMER1* (APC membrane recruitment protein 1) gene in craniofacial development using zebrafish as a model organism [13]. They utilised CRISPR/Cas9 to create an *AMER1* knockdown model in zebrafish. Craniofacial phenotypes were assessed using morphological analysis and molecular techniques, including qPCR, Western blotting, and immunofluorescence. To confirm the involvement of the Wnt/β-catenin pathway, the study employed pathway-specific inhibitors (IWR-1-endo) to test whether they could reverse the observed defects. Developmental processes, such as cranial neural crest cell proliferation and differentiation into chondrocytes, were closely monitored using imaging and gene expression analyses. They identified that *AMER1* played a role as an antagonist of the Wnt/β-catenin signalling pathway. The study also demonstrated that application of IWR-1-endo, a reversible inhibitor of Wnt/β-catenin signalling, partially rescued the abnormal craniofacial phenotype in *AMER1* knockdown zebrafish, indicating that *AMER1* modulates craniofacial development through its effects on Wnt signalling. Additionally, this research linked *AMER1* dysfunction to a possible mechanism for microtia–atresia in humans. The findings have significant implications for understanding the genetic and molecular basis of congenital craniofacial disorders, especially in microtia–atresia. By elucidating the role of *AMER1* and its interaction with Wnt/β-catenin signalling, the study provides potential targets for therapeutic interventions. The overall outcome from this work advances the understanding of genetic causes underlying craniofacial malformations and lays the groundwork for translational research aimed at developing treatments for microtia–atresia and related conditions.

### 2.3. Immunology and Autoimmune Disease

T-cell receptor (TCR) plays a central role in immune homeostasis, with disruptions linked to autoimmune diseases, cancer, and infections. Manolios and Pham et al. explored the non-antigenic modulation of the TCR Cβ-FG loop and its implications for T-cell signalling [14]. The study employed in silico docking simulations to predict small molecule interactions with the TCR FG loop. In vitro antigen presentation assays validated the signalling effects of these small molecules. Protein interaction experiments were conducted to understand the FG loop’s role in signal transduction. The authors found that small molecules binding to the TCR Cβ-FG loop can alter TCR signalling independently of antigen engagement. This modulation affected downstream signalling pathways, including calcium flux, cytokine production, and T-cell proliferation. Importantly, these effects were highly specific to the Cβ-FG loop, underscoring its critical role in TCR structural dynamics and function. The study provided proof-of-concept that the Cβ-FG loop can be selectively targeted to modulate immune responses without interfering with antigen recognition, minimising the risk of broad immunosuppression. This study reveals the Cβ-FG loop as a novel and specific target for modulating TCR signalling. By demonstrating that small molecules can influence immune responses without interfering with antigen recognition, the research highlights a pathway for developing highly targeted immunomodulatory therapies. The non-antigenic strategy represents a paradigm shift in how TCR signalling could be manipulated therapeutically and has broad implications for treating autoimmune diseases, cancers, and other conditions involving dysregulated immune responses.

### 2.4. Agricultural Biotechnology

The integration of translational bioinformatics and agricultural biotechnology can help to identify key molecular factors that enhance crop yield and nutritional value and reduce resistance to diseases, pests, and environmental stresses. Hou and Fan et al. explored the role of ribosome pausing in regulating protein translation during the transition of maize seedlings from dark-to-light conditions [15]. They employed Ribo-seq, a technique that allows single-codon resolution mapping of ribosome positions on mRNA. This was complemented by non-targeted proteomics to quantify changes in protein expression. Comparative analyses between dark and light conditions revealed the dynamics of ribosome pausing. The study identified over 400 ribosome-pausing events in etiolated maize seedlings, predominantly occurring in the dark and rapidly resolving upon light exposure. These events, linked to conserved nucleotide motifs, were shown to negatively regulate translation of specific genes, highlighting ribosome pausing as a key mechanism in gene expression control during light-induced growth transitions. The knowledge of translational regulatory mechanisms during dark-to-light transitions in maize seedlings gained from this study could be applied to improve crop yields, develop plants more resilient to changing environments, and optimise photosynthetic efficiency during growth transitions.

### 2.5. Human Mitochondrial DNA Database

Translational bioinformatics, combined with specialised databases focusing on human mitochondrial DNA, provides powerful tools for uncovering genetic variations, disease associations, and functional insights into mitochondrial biology. Shen-Gunther et al. sought to address the growing need for efficient and accurate mitochondrial DNA (mtDNA) analysis by developing a specialised database and bioinformatics workflow called hMITO DB v1.0 [16]. The authors designed a CLC Genomics workflow integrating the hMITO DB database for variant analysis, haplotyping, and geographic inference. Comparative analyses with existing tools highlighted the improved speed and accuracy of the workflow. The hMITO DB was built using a dataset of 4286 mitogenomes and integrated into a CLC Genomics workflow for automated read mapping, variant analysis, and haplotyping. The database produced accurate results consistent with published haplogroup distributions and phylogenetic trees. The authors tested the workflow on real-world samples using mtDNA-NGS sequences derived from Pap smears and cervical cancer cell lines to demonstrate their ability to generate comprehensive results for 47 samples in a short timeframe of 15 min. Compared to existing methods, the hMITO DB proved to be more efficient, reducing the computational burden while maintaining high accuracy. Additionally, the macrohaplogroup distribution and representative phylogenetic tree generated by the database aligned with established mitochondrial research, validating its reliability. Furthermore, hMITO DB addresses critical limitations in current mtDNA analysis tools by providing a rapid, efficient, and user-friendly platform. The hMITO DB combines a comprehensive database with an automated workflow, enabling researchers to perform complex analyses in minutes. The alignment of its results with established data further underscores its accuracy and reliability. The study highlights the potential of hMITO DB to revolutionise mitochondrial genomics research by making it more accessible and scalable across diverse fields.

## 3. Broad Implications and Future Directions

The studies featured in this Special Issue highlight the transformative potential of translational bioinformatics in tackling pressing challenges and shaping the future of molecular sciences. By advancing precision medicine and elucidating the molecular underpinnings of complex biological processes, the research featured here demonstrates the extensive impact of the convergence of biological data with computational tools (i.e., machine learning and bioinformatics) that will redefine healthcare, agriculture, and environmental sustainability. Moreover, the translational potential of this work is profound, with implications for repurposing drugs, developing biomarkers, and enhancing clinical applications that improve health and quality of life. The rapid progress in molecular sciences, driven by technological innovation and interdisciplinary collaboration, paves the way for transformative breakthroughs, translating fundamental discoveries into practical applications that enhance human health and quality of life. Priorities for upcoming research include integrating multi-omics data for cohesive biological insights, leveraging explainable artificial intelligence models for transparency in clinical applications, fostering global data-sharing partnerships to ensure equitable resource access, and addressing ethical considerations in data privacy and gene-editing technologies.

## 4. Conclusions

This Special Issue showcases the significant impact of molecular sciences in addressing diverse challenges across industrial, biological, and clinical domains. By highlighting the different dynamics of cutting-edge research, the research features in this Special Issue serve as a platform to inspire further discoveries and innovations, highlighting the profound impact of interdisciplinary collaboration. The included studies range from innovative bioinformatics tools to groundbreaking cancer research and environmental molecular insights, reflecting the versatility and far-reaching applications of molecular science. This collection serves as a valuable resource for researchers, clinicians, and policymakers, reinforcing the significance of the contributions within. In the years ahead, the integration of translational bioinformatics will remain central to unlocking new frontiers in molecular science, driving progress, and fostering solutions to some of the world’s most pressing challenges.

## Data Availability

Not applicable.
